# Age-specific associations between serum cholesterol levels and suicidal behaviors in patients with depressive disorders: A naturalistic prospective observational cohort study

**DOI:** 10.3389/fpsyt.2023.1095579

**Published:** 2023-04-17

**Authors:** Wonsuk Choi, Hee-Ju Kang, Ju-Wan Kim, Hee Kyung Kim, Ho-Cheol Kang, Ju-Yeon Lee, Sung-Wan Kim, Robert Stewart, Jae-Min Kim

**Affiliations:** ^1^Department of Internal Medicine, Chonnam National University Hwasun Hospital, Chonnam National University Medical School, Hwasun, Republic of Korea; ^2^Department of Psychiatry, Chonnam National University Medical School, Gwangju, Republic of Korea; ^3^King’s College London, Institute of Psychiatry, Psychology and Neuroscience, London, United Kingdom; ^4^South London and Maudsley NHS Foundation Trust, London, United Kingdom

**Keywords:** age, suicide, prediction, depression, cholesterol

## Abstract

**Introduction:**

This study investigated the effects of total cholesterol levels on prevalent, and incident suicidal behaviors according to age group (<60 vs. ≥60 years) in depressed patients.

**Methods:**

Consecutive outpatients with depressive disorders who visited the Chonnam National University Hospital between March 2012 and April 2017 were recruited. Among 1,262 patients assessed at baseline, 1,094 agreed to blood sampling for measurement of serum total cholesterol levels. Among the patients, 884 completed the 12-week acute treatment phase and were followed up at least once during the 12-month continuation treatment phase. Suicidal behaviors assessed at baseline included baseline suicidal severity; behaviors assessed at the 1-year follow-up included increased suicidal severity and fatal/non-fatal suicide attempts. Associations of baseline total cholesterol levels with the above-mentioned suicidal behaviors were analyzed using logistic regression models after adjustment for relevant covariates.

**Results:**

Of 1,094 depressed patients, 753 (68.8%) were women. The mean (SD) age of patients was 57.0 (14.9) years. Lower total cholesterol levels (87–161 mg/dl) were associated with increased suicidal severity (Linear Wald = 4.478, *p* < 0.05) and fatal/non-fatal suicide attempt (Linear Wald = 7.490, *p* < 0.01) in patients <60 years of age. U-shaped associations between total cholesterol levels and 1-year follow-up suicidal outcomes (increased suicidal severity, Quadratic Wald = 6.299, *p* < 0.05; fatal/non-fatal suicide attempt, Quadratic Wald = 5.697, *p* < 0.05) were observed in patients ≥60 years of age.

**Conclusions:**

These findings suggest that differential consideration of serum total cholesterol levels according to age group may have clinical utility for predicting suicidality in patients with depressive disorders. However, because our research participants came from a single-hospital, the generalizability of our findings may be limited.

## 1. Introduction

Suicide is a global public health problem that contributes to approximately 800,000 deaths each year (1). The spectrum of suicidal behavior ranges from suicidal ideation (SI) and suicide attempts (non-fatal suicidal behaviors) to suicide completion (fatal suicidal behavior); non-fatal suicidal behaviors are more common than fatal suicidal behaviors (2). Patients with depressive disorders are at high risk of suicide (3). Thus, there is a need to develop a biomarker that can be used to evaluate the risk of suicide in depressed patients and establish a preventive intervention strategy.

Among biomarkers, total cholesterol levels have been extensively investigated for monitoring and prediction of suicide risk. In most studies, low total cholesterol levels were reportedly associated with greater risk of suicidal behaviors including SI (4), suicide attempt (5–8), and suicide completion (9–12). Furthermore, a meta-analysis revealed that suicidal patients’ total cholesterol levels were considerably lower than those of both non-suicidal patients and healthy controls (13). However, contradictory results have been reported in a small number of studies, including an association with lower risk of suicidal behavior (14–17) or no significant association (18–21). Previous studies mainly involved working age adults, while some studies included elderly individuals (4, 5, 8, 10, 11, 17, 19, 20).

In our previous study that involved only elderly Koreans, higher total cholesterol levels were associated with both prevalent SI assessed at baseline and incident SI assessed at the 2-year follow-up (22). These observations were explained by the association between higher total cholesterol levels and stroke, which is strongly associated with suicidal behaviors (23). Because the prevalence of stroke is significantly higher in elderly individuals than in younger individuals (24) and hypercholesterolemia is a risk factor for stroke (24), the effects of total cholesterol levels on suicidal behavior in elderly individuals may differ from the effects in younger individuals. Moreover, since depressed patients are a high-risk group for suicide (25), and elderly depressed patients have a high prevalence of silent stroke (26), the effect of total cholesterol levels on suicidal behaviors in depressed patients may differ significantly depending on age. However, the associations between total cholesterol levels and suicidal behaviors according to age group (young vs. elderly adults) in depressed patients have not been previously investigated.

Here, we used data from a prospective study of Korean patients with depressive disorders to investigate the association between total cholesterol levels and prevalent and incident suicidal behaviors according to age group.

## 2. Materials and methods

### 2.1. Patient and public involvement

This study was conducted as a post-hoc analysis of the MAKE Biomarker discovery for Enhancing anTidepressant Treatment Effect and Response (MAKE BETTER) program. The study content was published as a design paper (27) and the protocol was registered at cris.nih.go.kr (identifier: KCT0001332). Although pre-determined protocols were used to decide interventions, patient preferences were prioritized regarding medication types, dosages, and regimens. Protocols of pharmacotherapeutics administered in this study have been previously published (28). The sociodemographic and clinical information at baseline and treatment-related data at follow-up were collected—using a structured clinical report form—by clinical research coordinators blinded to the treatment regimens. The staff members were trained in clinical report form administration and data collection methods by research physicians. Patient data were updated to the MAKE BETTER website^1^ within 3 days and supervised by data management personnel at the research center. The study was approved by the Chonnam National University Hospital Institutional Review Board (approval no. CNUH 2012-014).

### 2.2. Pharmacological treatment

Prior to initiation of treatment, patient clinical symptoms (e.g., presence of psychotic or anxiety symptoms), disease severity, physical comorbidities, medication profiles, and history of previous treatments were comprehensively reviewed. Treatment for lowering cholesterol was not considered. The minimum and maximum doses of pharmacotherapeutics were determined in accordance with existing treatment guidelines (29, 30). In Step 1 treatment, antidepressant monotherapy was administered for 3 weeks, in accordance with patient data and existing treatment guidelines (30–32). The antidepressants used included bupropion, desvenlafaxine, duloxetine, escitalopram, fluoxetine, mirtazapine, paroxetine, sertraline, venlafaxine, and vortioxetine. After Step 1 (antidepressant monotherapy), “next-step” pharmacotherapy was administered as needed at 3-week intervals in the acute treatment phase (3, 6, 9, and 12 weeks) and at 3-month intervals in the continuation treatment phase (6, 9, and 12 months). At the end of each step, overall effectiveness and tolerability were reviewed before proceeding to the next step. In the event of insufficient improvement (HAMD score reduction <30%) or intolerable side effects, patients were permitted to either remain in the current step or progress to subsequent steps by switching to other antidepressants (S), taking additional drugs other than antidepressants (A), using other antidepressants in combination (C), or using multiple strategies (S + A; S + C; A + C; S + A + C). Patients were allowed to proceed to the next-step treatment regardless of whether they showed sufficient improvement (HAMD score reduction ≥30%) and lacked intolerable side effects. For determination of treatment strategies, patient preference was prioritized to maximize medication compliance and treatment outcomes. The switched or combined antidepressants included bupropion, desvenlafaxine, duloxetine, escitalopram, fluoxetine, mirtazapine, paroxetine, sertraline, venlafaxine, and vortioxetine. The augmentation drugs were buspirone, lithium, triiodothyronine, and atypical antipsychotics (e.g., aripiprazole, risperidone, olanzapine, quetiapine, and ziprasidone). Because few patients progressed to Step 5 or above, treatment steps were reclassified as Steps 1–4 (including Step 5 and onward) for analysis purposes. Medication adherence was estimated based on the tablet counts at every visit, and was defined as poor in patients with <50% intake (33).

### 2.3. Participants

Consecutive outpatients with depressive disorders who visited the Chonnam National University Hospital psychiatric department between March 2012 and April 2017 and satisfied the eligibility criteria (Supplementary methods) were recruited. All included patients were newly treated cases, regardless of whether they experienced new-onset or recurrent depressive episodes. Because the primary aim in the MAKE BETTER study was to identify predictive biomarkers for short- and long-term pharmacotherapeutic outcomes, all study participants received only antidepressant-based therapy after informed consent had been obtained.

### 2.4. Exposure variables

#### 2.4.1. Serum total cholesterol levels

Participants were instructed to fast the night before the morning blood draw, then asked to relax for 25–45 min before sample collection. Serum total cholesterol levels were measured once in the baseline using the L-Type CHO M cholesterol oxidase method kit (Wako Pure Chemical Industries, Osaka, Japan) at the Global Clinical Central Lab (Yongin, Korea). The serum total cholesterol levels were divided into tertiles: high (195–485 mg/dl), middle (162–194 mg/dl), and low (87–161 mg/dl).

#### 2.4.2. Age

Age at baseline was recorded. To investigate the effects of baseline total cholesterol levels on suicidal behaviors between patients with late-life depression and others, participants were classified into groups according to age: <60 years of age and ≥60 years of age, as in previous studies (34).

### 2.5. Baseline covariates

The sociodemographic characteristics included age, sex, duration of formal education, marital status (currently married or not), cohabitation status (living alone or not), religion (religious observance or none), occupation (currently employed or not), monthly income (≥ or < 2,000 USD), and body mass index (BMI). The clinical characteristics assessed included diagnoses of depressive disorders with certain specifiers, age at onset, duration of illness, history of previous depressive episodes (recurrent or first episode), number of previous depressive episodes, duration of present episode, family history of depression, childhood abuse, number of concurrent physical disorders (evaluated using a questionnaire that included approximately 15 different systems/disorders), and number of stressful life events experienced by respondents during the 3 months prior to the interview (35). Assessment scales were used to investigate symptoms and functions as follows: depressive symptoms, Hamilton Depression Rating Scale (HAMD) (36); anxiety symptoms, Hospital Anxiety Depression Scale-anxiety subscale (HADS-A) (37); quality of life, EuroQol-5D instrument (EQ-5D) (38); functioning status, Social and Occupational Functioning Assessment Scale (SOFAS); and subjective perceptions of stress, Perceived Stress Scale (PSS) (39). HAMD (40), EQ-5D (41), SOFAS (42), and PSS (43) scales were formally standardized and validated in Koreans.

### 2.6. Outcome measures

#### 2.6.1. Baseline suicidal severity

The Brief Psychiatric Rating Scale (BPRS) suicidality scale score was used to evaluate baseline suicidal severity. BPRS scale was formally standardized and validated in Koreans (44). Participants were asked “Have you felt that life wasn’t worth living?,” “Have you thought about harming or killing yourself?,” “Have you felt tired of living, as though you would be better off dead?,” and “Have you ever felt like ending it all?.” If participants reported SI, further questions were asked, including “How often have you thought about this?” and “Do you have a specific plan?.” Participant responses were graded from 1 to 7 and classified into low [scores of 1 (not present)–3 (mild)] and high [scores of 4 (moderate)–7 (extremely severe)] suicide severity groups.

#### 2.6.2. Increased suicidal severity

The BPRS suicidality scale score was reassessed during the 1-year follow-up period at 3, 6, 9, and 12 weeks and at 6, 9, and 12 months. An increase in score during the follow-up period was defined as increased suicidal severity.

#### 2.6.3. Fatal and non-fatal suicide attempt

During the 1-year follow-up period, suicide attempts were recorded, which were defined as self-reported deliberate self-harm with at least some intention to die, regardless of the objective lethality (45). According to family source information, a fatal suicide attempt was discovered.

### 2.7. Statistical analysis

Patient baseline data were compared according to their serum total cholesterol levels (low vs. middle vs. high) and age (<60 vs. ≥60 years) using the independent *t*-test or chi-squared test. Unadjusted associations of baseline total cholesterol levels with three types of suicidal behaviors were investigated using the chi-squared test for the ternary variable (low vs. middle vs. high) and independent *t*-test for the continuous variable. Associations of total cholesterol levels with three types of suicidal behaviors were analyzed using logistic regression after adjustment for relevant covariates. Associations were tested by entering both linear (high group as the reference category) and non-linear quadratic terms for ternary variables of total cholesterol levels. Association strengths were estimated as Wald coefficients for both linear and quadratic terms. Interaction effects of serum total cholesterol levels (low vs. middle vs. high) and age (<60 vs. ≥60 years) on the suicidal behaviors were analyzed using logistic regression after adjusting for potential covariates. All statistical tests were two-sided with a significance level of 0.05. Bonferroni correction was used to maintain an overall type 1 error rate of 0.05 for 6 comparisons. A two-sided *p*-value of 0.008 (0.05/6) was taken to indicate statistical significance. Statistical analyses were performed using SPSS Statistics software (ver. 25.0; IBM Corp., Armonk, NY, United States) and STATA Statistics software (ver. 12.0; StataCorp, College Station, TX, United States).

## 3. Results

### 3.1. Recruitment and flow

**Figure 1:**
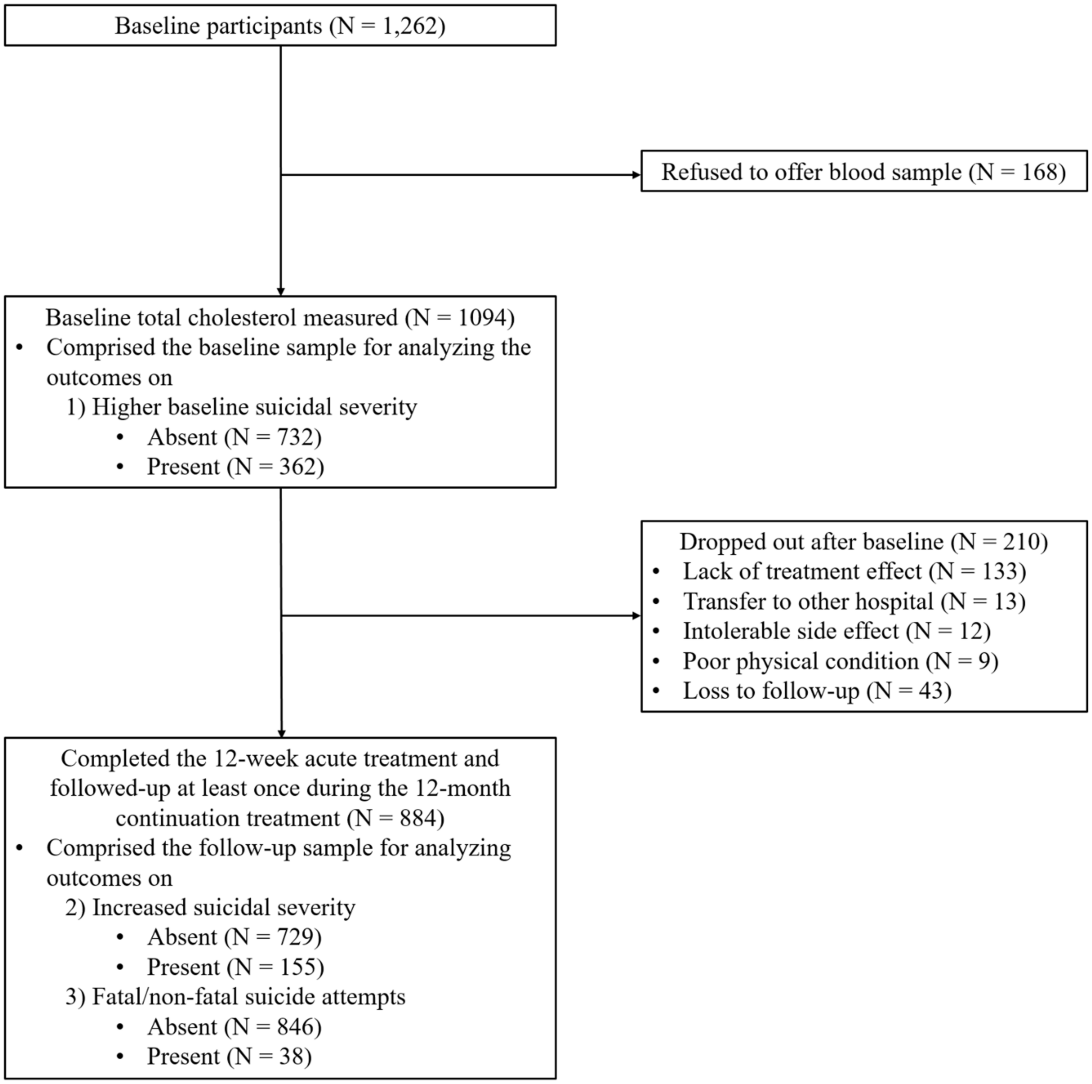
Participant recruitment and flow.

Patient recruitment and flow are shown in Figure 1. Among 1,262 patients assessed at baseline, 1,094 (86.7%) agreed to blood sampling for measurement of serum total cholesterol levels (including the baseline sample). Among the patients, 884 (80.8%) completed the 12-week acute treatment phase and were followed up at least once during the 12-month continuation treatment phase (follow-up sample). Reasons for drop-out included ineffective treatment (*N* = 133), transfer to other hospital (*N* = 13), intolerable side effects (*N* = 12), poor physical condition (*N* = 9), and loss to follow-up (*N* = 43). Significant differences were not found in baseline characteristics between patients with and without blood samples. However, drop-out at 12 months was significantly associated with unemployment and melancholic features at baseline.

### 3.2. Baseline characteristics

Comparisons of baseline characteristics according to serum total cholesterol levels in the baseline sample are shown in Table 1. Higher total cholesterol levels were significantly associated with female sex, lower educational level, higher BMI, lower frequency of family history of depression, lower frequency of childhood abuse, lower number of physical disorders, and higher HADS-A scores. Comparison of baseline characteristics according to age is shown in Supplementary Table 1. Age ≥ 60 years was significantly associated with lower educational level, living alone, religious status, unemployment, monthly income <2,000 USD, melancholic features, lower frequency of atypical features, higher age at onset, longer duration of illness, lower frequency of recurrent depression, lower number of depressive episodes, lower frequency of family history of depression, lower frequency of childhood abuse, higher number of physical disorders, lower number of stressful life events, lower HADS-A scores, and lower PSS scores. Based on the statistical significance (*p* < 0.05) and potential for multicollinearity, the following covariates were selected for subsequent adjusted analyses: sex, educational level, living alone, religious observance, BMI, melancholic features, atypical features, duration of illness, recurrent depression, family history of depression, childhood abuse, number of physical disorders, and HADS-A and PSS scores.

**Table 1:** Baseline characteristics according to serum total cholesterol levels (*N* = 1,094).

	Low-total cholesterol (*N* = 367)	Middle-total cholesterol (*N* = 361)	High-total cholesterol (*N* = 366)	Statistical coefficients^a^	*p*-value
Socio-demographic characteristics					
Age, mean (SD) years	55.8 (16.5)	56.9 (14.4)	58.2 (13.4)	*F* = 2.357	0.095
Sex, N (%) female	226 (61.6)	238 (65.9)	289 (79.0)	*χ*^2^ = 27.920	< 0.001
Education, mean (SD) years	9.6 (4.6)	9.3 (4.7)	8.4 (5.0)^b^	*F* = 5.687	0.003
Marital status, N (%) unmarried	107 (29.2)	110 (30.5)	110 (30.1)	*χ*^2^ = 0.157	0.924
Living alone, N (%)	46 (12.5)	64 (17.7)	57 (15.6)	*χ*^2^ = 3.837	0.147
Religious observance, N (%)	197 (53.7)	204 (56.5)	210 (57.4)	*χ*^2^ = 1.112	0.574
Unemployed status, N (%)	104 (28.3)	108 (29.9)	104 (28.4)	*χ*^2^ = 0.280	0.869
Monthly income, N (%) <2,000 USD	226 (61.6)	211 (58.4)	216 (59.0)	*χ*^2^ = 0.845	0.655
Body mass index, mean (SD) kg/m^2^	22.8 (3.2)	23.2 (2.9)	23.7 (3.3)^b^	*F* = 6.395	0.002
Clinical characteristics					
Major depressive disorder, N (%)	310 (84.5)	313 (86.7)	310 (84.7)	*χ*^2^ = 0.874	0.646
Melancholic feature, N (%)	47 (12.8)	60 (16.6)	58 (15.8)	*χ*^2^ = 2.318	0.314
Atypical feature, N (%)	29 (7.9)	23 (6.4)	17 (4.6)	*χ*^2^ = 3.294	0.193
Age at onset, mean (SD) years	51.1 (17.9)	51.5 (16.6)	53.1 (15.5)	*F* = 1.440	0.237
Duration of illness, mean (SD) years	4.8 (8.9)	5.3 (9.0)	5.1 (9.3)	*F* = 0.395	0.674
Recurrent depression, N (%)	192 (52.3)	190 (52.6)	191 (52.2)	χ^2^ = 0.015	0.992
Number of depressive episodes, mean (SD)	1.1 (1.4)	1.2 (1.6)	1.0 (1.4)	*F* = 0.957	0.384
Duration of present episode, mean (SD) months	7.5 (9.7)	6.8 (10.2)	7.9 (11.1)	*F* = 0.875	0.417
Family history of depression, N (%)	68 (18.5)	46 (12.7)	46 (12.6)	*χ*^2^ = 6.743	0.034
Any childhood abuse, N (%) present	63 (17.2)	47 (13.0)	39 (10.7)	*χ*^2^ = 6.767	0.034
Number of physical disorders, mean (SD)	1.8 (1.3)	1.6 (1.2)	1.5 (1.2)^b^	*F* = 4.784	0.009
Number of stressful life events, mean (SD)	2.1 (1.9)	2.0 (1.4)	1.9 (1.2)	*F* = 0.827	0.438
Assessment scales, mean (SD) scores					
Hamilton Depression Rating Scale	20.6 (4.1)	20.8 (4.3)	20.9 (4.1)	*F* = 0.433	0.649
Hospital Anxiety & Depression Scale-anxiety subscale	11.2 (4.1)	12.1 (4.0)^b^	12.0 (4.0)^b^	*F* = 5.611	0.004
EuroQol-5D	8.9 (1.5)	8.9 (1.5)	9.0 (1.5)	*F* = 0.131	0.877
Social and Occupational Functional Assessment Scale	56.0 (7.3)	55.8 (7.5)	56.1 (7.6)	*F* = 0.138	0.871
Perceived Stress Scale, mean	26.9 (6.5)	27.3 (6.6)	27.0 (6.5)	*F* = 0.365	0.695
Treatment step over 1 year (*N* = 884), *N* (%)					
Step 1	147 (40.1)	144 (39.9)	149 (40.7)	*χ*^2^ = 5.098	0.531
Step 2	103 (28.1)	118 (32.7)	124 (33.9)		
Step 3	79 (21.5)	66 (18.3)	63 (17.2)		
Step 4	38 (10.4)	33 (9.1)	30 (8.2)		

a Analysis of variance test or *χ*^2^ test, as appropriate.

b *p* < 0.05 vs. low cholesterol in post hoc analysis.

### 3.3. Unadjusted associations of serum total cholesterol levels with suicidal behaviors

Comparisons of baseline serum total cholesterol levels between participants with and without the four types of suicidal behaviors are shown in Supplementary Table 2. The middle-total cholesterol group showed the lowest frequencies of suicidal behaviors evaluated at the 1-year follow-up (increased suicidal severity and fatal/non-fatal suicide attempt). However, the frequencies of suicidal behaviors evaluated at baseline (higher baseline suicidal severity) did not significantly differ among the three groups. Total cholesterol levels showed no differences between patients with and without higher baseline suicidal severity, increased suicidal severity, and fatal/non-fatal suicide attempt.

### 3.4. Adjusted associations of serum total cholesterol levels with suicidal behaviors according to age group

**Figure 2:**
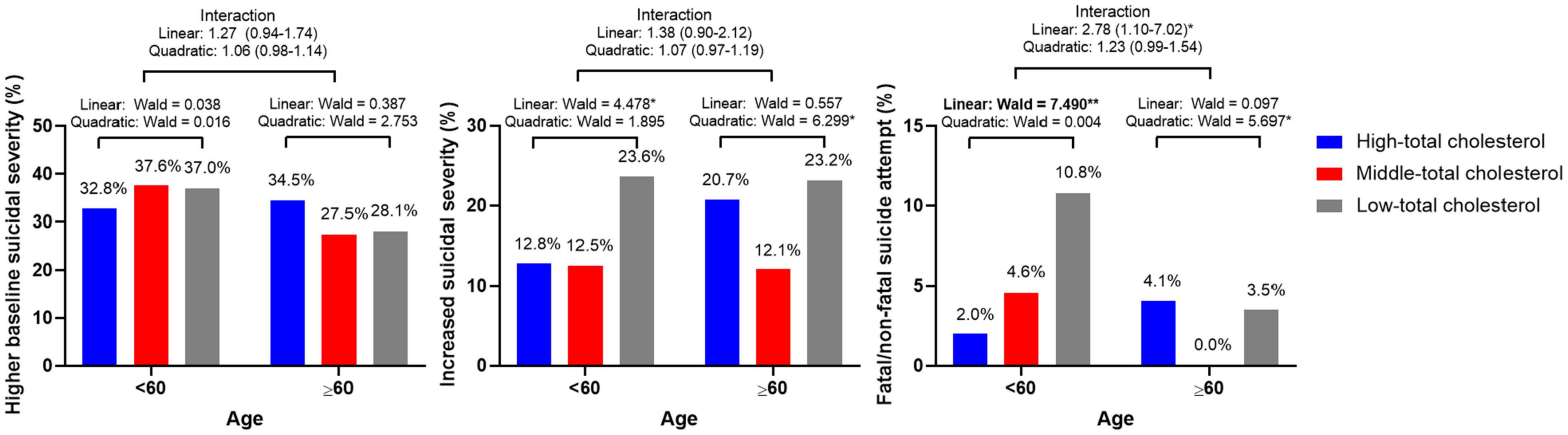
Adjusted linear and quadratic associations of serum cholesterol levels with suicidal behaviors according to age group. Figure legend: Data represent Wald scores or odds ratios (95% confidence intervals) after adjustment for sex, educational level, living alone, religious observance, body mass index (BMI), melancholic features, atypical features, duration of illness, recurrent depression, family history of depression, childhood abuse, number of physical disorders, and Hospital Anxiety Depression Scale-Anxiety Subscale (HADS-A) and Perceived Stress Scale (PSS) scores. ^*^p < 0.05 and ^**^p < 0.01. Bold style denotes statistical significance after bonferroni correction.

The effects of serum total cholesterol levels on suicidal behaviors according to age group (<60 vs. ≥60 years) are shown in Table 2. Among patients <60 years of age, the low-total cholesterol group showed a higher incidence of increased suicidal severity and fatal/non-fatal suicide outcome, compared with the high-total cholesterol group, after adjustment for relevant covariates. However, these effects were not observed in patients ≥60 years of age. The adjusted associations between serum total cholesterol levels and suicidal behaviors according to age group (<60 vs. ≥60 years) are shown in Figure 2. Among patients <60 years of age, lower total cholesterol levels were associated with increased suicidal severity and fatal/non-fatal suicide attempt. The linear terms for the associations of baseline total cholesterol levels with the two suicidal outcomes were significant after adjustment for relevant covariates. Among patients ≥60 years of age, U-shaped associations were observed between baseline total cholesterol levels and 1-year follow-up suicidal outcomes (increased suicidal severity and fatal/non-fatal suicide attempt). The quadratic terms for the associations of baseline total cholesterol levels with 1-year follow-up suicidal outcomes were significant after adjustment for relevant covariates. The interaction term was statistically significant only for linear associations between total cholesterol levels and fatal/non-fatal suicide attempt.

**Table 2:** Effects of serum total cholesterol levels on suicidal behaviors at baseline (N = 1,094) and follow-up (N = 884) according to age group.

Age	Total cholesterol (tertile)	At baseline (*N* = 1,094)			During 1-year follow-up (*N* = 884)				
		*N*	Higher baseline suicidal severity^a^		*N*	Increased suicidal severity^b^		Fatal/non-fatal suicide attempt	
			*N* (%)	Odds ratio (95% CI)^c^		*N* (%)	Odds ratio (95% CI)^c^	*N* (%)	Odds ratio (95% CI)^c^
<60	High	189	62 (32.8)	Reference	148	19 (12.8)	Reference	3 (2.0)	Reference
	Middle	194	73 (37.6)	1.05 (0.66–1.68)	152	19 (12.5)	0.93 (0.46–1.89)	7 (4.6)	2.36 (0.55–10.06)
	Low	189	70 (37.0)	1.05 (0.64–1.73)	157	37 (23.6)	1.99 (1.02–3.88)^*^	17 (10.8)	5.95 (1.51–23.53)^*^
≥60	High	177	61 (34.5)	Reference	145	30 (20.7)	Reference	6 (4.1)	Reference
	Middle	167	46 (27.5)	0.63 (0.37–1.06)	140	17 (12.1)	0.52 (0.27–1.03)	0 (0.0)	0.00 (N/A)
	Low	178	50 (28.1)	0.86 (0.51–1.45)	142	33 (23.2)	1.27 (0.68–2.37)	5 (3.5)	0.96 (0.23–4.02)

a Brief Psychiatric Rating Scale suicidality scale score of 4 (moderate)–7 (extremely severe).

b Increase in Brief Psychiatric Rating Scale suicidality item score during follow-up, compared with baseline.

c Adjusted for sex, educational level, living alone, religious observance, body mass index (BMI), melancholic features, atypical features, duration of illness, recurrent depression, family history of depression, childhood abuse, number of physical disorders, and Hospital Anxiety Depression Scale-Anxiety Subscale (HADS-A) and Perceived Stress Scale (PSS) scores.

* *p* < 0.05 (All *p*-values are empirical since the approach was not really “hypothesis free” and based on previous evidence on the associations).

## 4. Discussion

In the present study, which used data from a prospective study that reflected the real-world clinical setting, different associations between baseline serum total cholesterol levels and suicidal behaviors were investigated according to age group (<60 vs. ≥60 years) in depressed patients. In patients <60 years of age, lower total cholesterol levels were associated with 1-year follow-up suicidal outcomes. In patients ≥60 years of age, U-shaped associations between total cholesterol levels and 1-year follow-up suicidal outcomes were observed. Both lower and higher total cholesterol levels exhibited significant associations with 1-year follow-up suicidal outcomes; these associations remained robust after adjustment for relevant covariates.

Many clinical studies have been conducted to investigate the associations between total cholesterol levels and suicide risk. In multiple studies, lower total cholesterol levels were associated with increased risk of suicide (4–12). Similar findings were also found in a meta-analysis that included the outcomes of SI, suicide attempts, and suicide completion (13). Our findings concerning patients <60 years of age were largely consistent with the previously reported observations. These associations can be explained based on the cholesterol-serotonin hypothesis: low cholesterol levels downregulate serotonergic activity in the central nervous system, leading to aggressive behavior in vulnerable individuals (46, 47).

However, some studies of adults have shown that an increased risk of suicide is associated with higher total cholesterol levels (14–17). Because higher total cholesterol levels are a risk factor for stroke (24) and the incidence of suicidal behaviors is increased after stroke (23), this association was presumed to be a secondary change in behavior after stroke. In addition, a study of community-dwelling elderly individuals demonstrated that higher total cholesterol levels were associated with both prevalent and incident SI (22). Similarly, in patients ≥60 years of age in the present study, U-shaped associations between total cholesterol levels and 1-year follow-up suicidal outcomes were observed without any significant linear associations, indicating that both higher and lower baseline total cholesterol levels may predict future suicidal outcomes. Because aging is an independent cardiovascular risk factor in adults (48) and a significant portion of patients with late-life depression have silent stroke (26), higher total cholesterol levels may be selectively associated with suicidal behaviors in elderly individuals.

Notably, U-shaped associations between total cholesterol levels and suicidal behaviors were observed only for prospective suicidal outcomes. These results were presumably related to patient classification into high or low total cholesterol groups according to total cholesterol levels at baseline. Because the effects of altered total cholesterol levels on suicidal behaviors are likely to be secondary changes, long-term exposure to altered total cholesterol levels might only be associated with prospective suicidal behaviors. However, because participants’ total cholesterol levels were not evaluated during the study period, further investigation is needed. Additionally, given that baseline suicidal behavior was measured in depressed individuals who had never received antidepressant treatment, disease-related characteristics may have had a greater influence. After treating the disease-related factors with antidepressant medication, the effects of total cholesterol levels on suicidal behavior may have been identified.

As stated above, studies concerning the associations between total cholesterol levels and suicidal behaviors have been controversial with positive relationships (14–17), negative relationships (5–12), and no relationship reported (18–21). Our findings showed that associations between total cholesterol levels and suicidal behaviors differed according to age group; these may explain the heterogenous results in previous studies. The differential effects of total cholesterol levels on suicidal behaviors according to age group in our cohort are plausible for several reasons. First, decreased cholesterol levels reportedly increase the cellular membrane fluidity of serotonin receptor 1A (HTR1A) in the central nervous system (49) and downregulate HTR1A exposure in the synaptic cleft (50). These biological processes induced by lower total cholesterol levels may decrease serotonergic neurotransmission in the central nervous system, thus increasing the suicidal behavior in susceptible individuals (e.g., depressed patients in all age groups). Second, hypercholesterolemia is a well-known risk factor for stroke (24), and stroke is both prevalent in elderly individuals (24) and associated with suicide (23); therefore, higher total cholesterol levels may be selectively associated with suicidal behaviors only in elderly individuals. Because the present study only included patients who had been diagnosed with depressive disorders, which are risk factors for stroke (51), higher total cholesterol levels may contribute to stroke through a synergistic effect with depression, which may serve as a secondary factor increasing the risk of suicidal behaviors.

Depressed patients have a high risk of suicide (3). In previous studies, the associations between total cholesterol levels and suicidal behaviors in depressed patients have been investigated using cross-sectional case–control designs, comparing total cholesterol levels between participants with and without previous suicide attempt (5–8, 14, 20) or present SI (18). A particular strength of the present study was the hypothesis assessment using prospective suicidal behaviors. The present novel findings may have clinical utility in the prediction of future suicide risk in depressed patients. Other strengths include the large sample size, participant assessments using standardized questionnaires and scales, and consideration of multiple covariates that could affect the study results.

Several limitations should also be considered when interpreting the findings. First, associations between treatment-related changes in total cholesterol levels and prospective suicidal behaviors could not be evaluated because total cholesterol levels were only measured once at baseline. Because cholesterol levels can affect suicidal behaviors by altering serotonergic activity in the central nervous system (49, 50), treatment-related changes in total cholesterol levels may influence future suicidal behaviors. Second, information was unavailable concerning the use of lipid-lowering medications, which could alter total cholesterol levels. Third, pharmacotherapeutic agents were determined according to patient preference guided by the clinician, rather than using a pre-determined protocol, because of the study design. Thus, inter-clinician variability may have affected the association of interest. However, therapeutic decisions were made without knowing total cholesterol levels; thus, inter-clinician variability presumably did not affect the outcomes. Fourth, the effects of total cholesterol levels on future suicidal outcomes could not be differentially assessed according to antidepressant type because of heterogeneity among pharmacological regimens. Fifth, substantial loss to follow-up occurred during the 1-year study period. Because more patients with poor clinical characteristics were lost to follow-up, the incidences of 1-year follow-up suicidal outcomes may have been underestimated. However, because Republic of Korea values personal information and data is anonymized in the public database, it was not able to ascertain whether there was an increase in suicidal behavior in patients who did not conduct follow-up measures. Sixth, the generalizability of our findings may be limited because our study individuals came from a single center in South Korea. Seventh, our findings have limitations because certain results lose significance when Bonferroni correction is employed, although the approach was not really “hypothesis free” and based on previous evidence on the associations between cholesterol levels and suicidality. Eighth, because suicidal behaviors over a year were uncommon, our findings may not possess much power. Ninth, since the precise moment at which each suicidal behavior took place was unknown, survival analysis was not conducted in this study. Tenth, because the baseline characteristics of study subjects relating to cardiovascular disease risk factors or stroke were not assessed, it was unable to determine how much these factors contributed to the U-shaped association between total cholesterol level and prospective suicidal behaviors in elderly depressive patients. Finally, despite adjusting for multiple covariates that potentially influence suicidal behaviors, our findings may have been influenced by variations in the groups’ baseline characteristics.

In conclusion, the associations of baseline total cholesterol levels with suicidal behaviors differed according to age group; lower total cholesterol levels were associated with 1-year follow-up suicidal outcomes in patients <60 years of age, and U-shaped associations were observed between total cholesterol levels and 1-year follow-up suicidal outcomes in patients ≥60 years of age. The results may explain inconsistent results regarding the associations between total cholesterol levels and suicidal behaviors reported in previous studies. Higher total cholesterol levels may be preferentially associated with suicidal behaviors in elderly individuals because aging is an independent cardiovascular risk factor and a considerable fraction of patients with late-life depression suffer silent stroke. Our findings may be useful for the assessment of suicide risk in depressed patients and the establishment of preventive intervention strategies. However, further studies are needed to validate our findings.

## Data availability statement

The original contributions presented in the study are included in the article/Supplementary material, further inquiries can be directed to the corresponding author.

## Ethics statement

The studies involving human participants were reviewed and approved by Chonnam National University Hospital Institutional Review Board. The patients/participants provided their written informed consent to participate in this study.

## Author contributions

WC and J-MK: conceptualization, data curation, formal analysis, and writing. H-JK: data curation, methodology, and writing. J-WK: formal analysis, methodology, and writing. HK, H-CK, J-YL, and S-WK: data curation, validation, and project administration. RS: conceptualization, formal analysis, and writing. All authors contributed to the article and approved the submitted version.

## Funding

The study was funded by a grant of National Research Foundation of Korea Grant [NRF-2020M3E5D9080733 and NRF-2020R1A2C2003472] to J-MK. RS is part-funded by the National Institute for Health Research (NIHR) Biomedical Research Centre at South London and Maudsley NHS Foundation Trust and King’s College London, and from the National Institute for Health Research (NIHR) Applied Research Collaboration South London (NIHR ARC South London) at King’s College Hospital NHS Foundation Trust. RS is also a National Institute for Health Research (NIHR) Senior Investigator.

## Conflict of interest

J-MK declares research support in the last 5 years from Janssen and Lundbeck. RS declares research support in the last 5 years from Roche, Janssen, GSK, and Takeda. S-WK declares research support in the last 5 years from Janssen, Boehringer Ingelheim, Allergan and Otsuka.

The remaining authors declare that the research was conducted in the absence of any commercial or financial relationships that could be construed as a potential conflict of interest.

## Publisher’s note

All claims expressed in this article are solely those of the authors and do not necessarily represent those of their affiliated organizations, or those of the publisher, the editors and the reviewers. Any product that may be evaluated in this article, or claim that may be made by its manufacturer, is not guaranteed or endorsed by the publisher.

## Footnotes

^1^ http://icreat.nih.go.kr/icreat/webapps/com/hismainweb/jsp/cdc_n2.live
