# Supplementary material for: Age-specific associations between serum cholesterol levels and suicidal behaviors in patients with depressive disorders: A naturalistic prospective observational cohort study
Source: Front Psychiatry. 2023 Apr 17;14:1095579. doi: 10.3389/fpsyt.2023.1095579 (PMC10150039; doi:10.3389/fpsyt.2023.1095579)
Supplement: Supplementary file 1 [file Data_Sheet_1.docx]

**Supplementary Methods**

**Eligibility criteria**

The inclusion criteria were as follows: age > 7 years; diagnosis with major depressive disorder, dysthymic disorder, or depressive disorder not otherwise specified using the Mini-International Neuropsychiatric Interview (1), a structured diagnostic psychiatric interview based on the Diagnostic and Statistical Manual of Mental Disorders, Fourth Edition (DSM-IV) criteria; Hamilton Depression Rating Scale (HAMD) (2) score ≥ 14; and ability to complete questionnaires, understand the objective of the study, and provide informed consent. The exclusion criteria were as follows: unstable or uncontrolled medical conditions; inability to complete the psychiatric assessment or comply with the medication regimen because of severe physical illness; current or lifetime DSM-IV diagnosis of bipolar disorder, schizophrenia, schizoaffective disorder, schizophreniform disorder, psychotic disorder not otherwise specified, or other psychotic disorders; history of organic psychosis, epilepsy, or seizure disorders; history of anticonvulsant treatment; hospitalization for any psychiatric condition except depressive disorders (e.g., alcohol/drug dependence); electroconvulsive therapy for the current depressive episode; and pregnancy or breastfeeding. Written informed consent was obtained from all adult participants. For participants < 16 years of age, written consent was obtained from a parent or legal guardian and written assent was obtained from the participant.

**References**

1. Sheehan DV, Lecrubier Y, Sheehan KH, Amorim P, Janavs J, Weiller E, et al. The Mini-International Neuropsychiatric Interview (M.I.N.I.): The Development and Validation of a Structured Diagnostic Psychiatric Interview for Dsm-Iv and Icd-10. *J Clin Psychiatry* (1998) 59 Suppl 20:22-33;quiz 4-57. Epub 1999/01/09.

2. Hamilton M. A Rating Scale for Depression. *J Neurol Neurosurg Psychiatry* (1960) 23:56-62. Epub 1960/02/01. doi: 10.1136/jnnp.23.1.56.

| **Supplementary Table 1**  Baseline characteristics according to age (N = 1,094). | | | | | |
| --- | --- | --- | --- | --- | --- |
|  |  | <60 years (N = 572) | ≥60 years (N = 522) | Statistical coefficients^a^ | P-value |
| **Socio-demographic characteristics** |  |  |  |  |  |
| Sex, N (%) female |  | 390 (68.2) | 363 (69.5) | χ^2^ = 0.235 | 0.628 |
| Education, mean (SD) years |  | 11.4 (3.7) | 6.5 (4.5) | t = 19.502 | < 0.001 |
| Marital status, N (%) unmarried |  | 179 (31.3) | 148 (28.4) | χ^2^ = 1.127 | 0.288 |
| Living alone, N (%) |  | 61 (10.7) | 106 (20.3) | χ^2^ = 19.617 | < 0.001 |
| Religious observance, N (%) |  | 289 (50.5) | 322 (61.7) | χ^2^ = 13.789 | < 0.001 |
| Unemployed status, N (%) |  | 135 (23.6) | 181 (34.7) | χ^2^ = 16.291 | < 0.001 |
| Monthly income, N (%) <2,000 USD |  | 235 (41.1) | 418 (80.1) | χ^2^ = 172.464 | < 0.001 |
| Body mass index, mean (SD) kg/m^2^ |  | 23.1 (3.4) | 23.4 (3.0) | t = -1.787 | 0.074 |
| **Clinical characteristics** |  |  |  |  |  |
| Major depressive disorder, N (%) |  | 486 (85.0) | 447 (85.6) | χ^2^ = 0.097 | 0.756 |
| Melancholic feature, N (%) |  | 73 (12.8) | 92 (17.6) | χ^2^ = 5.038 | 0.025 |
| Atypical feature, N (%) |  | 56 (9.8) | 13 (2.5) | χ^2^ = 24.611 | < 0.001 |
| Age at onset, mean (SD) years |  | 41.6 (13.0) | 63.2 (12.5) | t = -27.987 | < 0.001 |
| Duration of illness, mean (SD) years |  | 4.4 (7.1) | 5.9 (10.7) | t = -2.698 | 0.007 |
| Recurrent depression, N (%) |  | 321 (56.1) | 252 (48.3) | χ^2^ = 6.731 | 0.009 |
| Number of depressive episodes, mean (SD) |  | 1.2 (1.6) | 0.9 (1.3) | t = 3.163 | 0.002 |
| Duration of present episode, mean (SD) months |  | 7.3 (10.2) | 7.4 (10.6) | t = -0.146 | 0.884 |
| Family history of depression, N (%) |  | 101 (17.7) | 59 (11.3) | χ^2^ = 8.827 | 0.003 |
| Any childhood abuse, N (%) present |  | 109 (19.1) | 40 (7.7) | χ^2^ = 30.113 | < 0.001 |
| Number of physical disorders, mean (SD) |  | 1.2 (1.2) | 2.1 (1.3) | t = -11.329 | < 0.001 |
| Number of stressful life events, mean (SD) |  | 2.3 (1.8) | 1.7 (1.1) | t = 6.936 | < 0.001 |
| Serum total cholesterol level, mean (SD) mg/dL |  | 181.2 (40.6) | 180.1 (40.7) | t = 0.451 | 0.652 |
| **Assessment scales, mean (SD) scores** |  |  |  |  |  |
| Hamilton Depression Rating Scale |  | 20.8 (4.2) | 20.7 (4.1) | t = 0.180 | 0.857 |
| Hospital Anxiety & Depression Scale-anxiety subscale |  | 12.3 (4.2) | 11.2 (3.8) | t = 4.713 | < 0.001 |
| EuroQol-5D |  | 8.9 (1.5) | 9.0 (1.6) | t = -1.520 | 0.129 |
| Social and Occupational Functional Assessment Scale |  | 55.6 (7.3) | 56.4 (7.6) | t = -1.819 | 0.069 |
| Perceived Stress Scale |  | 27.7 (6.5) | 26.3 (6.5) | t = 3.645 | < 0.001 |
| **Treatment step over 1 year (N = 884), N (%)** |  |  |  |  |  |
| Step 1 |  | 229 (40.0) | 211 (40.4) | χ^2^ = 6.517 | 0.089 |
| Step 2 |  | 169 (29.5) | 176 (33.7) |  |  |
| Step 3 |  | 110 (19.2) | 98 (18.8) |  |  |
| Step 4 |  | 64 (11.2) | 37 (7.1) |  |  |

^a^Independent two-sample *t*-test or χ^2^ test, as appropriate

| **Supplementary Table 2**  Unadjusted associations of serum total cholesterol levels with suicidal behaviors at baseline (N = 1,094) and during the 1-year follow-up (N = 884). | | | | | | | | | | | | | |
| --- | --- | --- | --- | --- | --- | --- | --- | --- | --- | --- | --- | --- | --- |
| Total cholesterol |  | At baseline (N = 1,094) | | | | During 1-year follow-up (N = 884) | | | | | | | |
|  |  | Higher baseline suicidal severity^a^ | | | | Increased suicidal severity^b^ | | | | Fatal/non-fatal suicide attempt | | | |
|  |  | Absent  (N = 732) | Present  (N = 362) | Statistical coefficient^c^ | P-value | Absent  (N = 729) | Present  (N = 155) | Statistical coefficient^c^ | P-value | Absent  (N = 846) | Present  (N = 38) | Statistical coefficient^c^ | P-value |
| High, N (%) |  | 243 (66.4) | 123 (33.6) | χ^2^ = 0.072 | 0.965 | 244 (83.3) | 49 (16.7) | χ^2^ = 12.748 | 0.002 | 284 (96.9) | 9 (3.1) | χ^2^ = 10.440 | 0.005 |
| Middle, N (%) |  | 242 (67.0) | 119 (33.0) |  |  | 256 (87.7) | 36 (12.3) |  |  | 285 (97.6) | 7 (2.4) |  |  |
| Low, N (%) |  | 247 (67.3) | 120 (32.7) |  |  | 229 (76.6) | 70 (23.4) |  |  | 277 (92.6) | 22 (7.4) |  |  |
|  |  |  |  |  |  |  |  |  |  |  |  |  |  |
| Mean (SD) mg/dL |  | 179.9 (39.0) | 182.2 (43.7) | t = -0.914 | 0.361 | 180.9 (36.5) | 176.3 (46.6) | t = 1.139 | 0.256 | 180.5 (40) | 171.6 (53.9) | t = 0.999 | 0.324 |

^a^Brief Psychiatric Rating Scale suicidality scale score of 4 (moderate)–7 (extremely severe)

^b^Increase in Brief Psychiatric Rating Scale suicidality item score during follow-up, compared with baseline

^c^χ^2^ test or independent two-sample *t*-test, as appropriate
